# MiR-7-5p/KLF4 signaling inhibits stemness and radioresistance in colorectal cancer

**DOI:** 10.1038/s41420-023-01339-8

**Published:** 2023-02-02

**Authors:** Yuanyuan Shang, Zhe Zhu, Yuanyuan Zhang, Fang Ji, Lian Zhu, Mengcheng Liu, Yewei Deng, Guifen Lv, Dan Li, Zhuqing Zhou, Bing Lu, Chuan-gang Fu

**Affiliations:** 1 Department of Colorectal Surgery, Department of General Surgery, Shanghai East Hospital, School of Medicine, Tongji University, Shanghai, 200120 China; 2 Department of Radiation Oncology, Shanghai East Hospital, School of Medicine, Tongji University, Shanghai, 200120 China

**Keywords:** Cancer therapeutic resistance, Cancer

## Abstract

Resistance to radiotherapy remains a major unmet clinical obstacle in the treatment of locally advanced rectal cancer. Cancer stem cells (CSCs) are considered to mediate tumor development and radioresistance. However, the role of CSCs in regulating resistance to radiotherapy in colorectal cancer (CRC) remains largely unknown. We established two radioresistant CRC cell lines, HCT116-R and RKO-R, using fractionated irradiation. Analysis using miRNA sequencing and quantitative real-time PCR confirmed lower levels of miR-7-5p in both of the radioresistant cells compared to their parental cells. Subsequently, we validated that miR-7-5p expression was decreased in cancerous tissues from radiotherapy-resistant rectal cancer patients. The Cancer Genome Atlas (TCGA) database analyses revealed that low miR-7-5p expression was significantly correlated with poor prognosis in CRC patients. Overexpression of miR-7-5p led to a rescue of radioresistance and an increase in radiation-induced apoptosis, and attenuated the stem cell-like properties in HCT116-R and RKO-R cells. Conversely, knocking down miR-7-5p in parental HCT116 and RKO cells suppressed the sensitivity to radiation treatment and enhance cancer cell stemness. Stemness-associated transcription factor KLF4 was demonstrated as a target of miR-7-5p. Rescue experiments revealed that miR-7-5p/KLF4 axis could induce radiosensitivity by regulating CSCs in colorectal cancer cells. Furthermore, we used CRC tumor tissues which exhibited resistance to neoadjuvant radiotherapy to establish a patient-derived xenograft (PDX) mouse model. Tail vein injection of magnetic nanoparticles carrying miR-7-5p mimics into the PDX mice significantly inhibited tumor growth with or without irradiation treatment in vivo. Our current studies not only demonstrate an anti-cancer function of miR-7-5p in regulating CSC properties and radiosensitivity in colorectal cancer, but also provide a novel potential strategy for delaying or reverse radiation resistance in preoperative radiotherapy of CRC patients.

## Introduction

Colorectal cancer (CRC) ranks the third most frequently diagnosed malignances and is responsible for ~10% of all cancer-associated mortality globally [1–3]. For locally advanced rectal cancer (LARC), the most common type of rectal cancer, a combination of neoadjuvant chemoradiotherapy (CRT) and total mesorectal excision (TME) operation is currently considered as a standard treatment regimen [4, 5]. However, due to varying degrees of sensitivity to chemo- and radiotherapy among individual patients, the response to neoadjuvant CRT displays large heterogeneity, ranging from complete to none [6–8]. Further understanding of the key molecular basis and signaling pathways predisposing the resistance to radiotherapy yields new potential predictive and therapeutic targets that will provide us with strategies on how to improve the therapeutic response and obtain better outcomes.

Cancer stem cells (CSCs), also known as tumor initiating cells (TICs) or stem-like cancer cells, account for a minor subpopulation of tumor cells, which are prominently characterized by enhanced tumorigenic, self-renewal and tumor-regeneration capacities [9]. The existence of CSCs has been identified in almost all types of human cancer, including leukemia [10], breast cancer [11], colorectal cancer [12], cervical cancer [13] and other malignancies. Accumulating evidence has revealed that CSCs are responsible for the resistance to therapeutic strategies, such as chemotherapy [14] and radiotherapy [15, 16]. In light of that, exploration of factors affecting the functions of CSCs and development of specific molecules targeting CSCs will ultimately enhance the radiotherapeutic efficacy and hence improve the outcomes of patients with LARC. Until now, several CSC markers like CD133, CD44, Lgr5 and EpCAM have been used to identify and isolate colorectal CSCs from tumor tissues [17]. However, limited information exists regarding colorectal CSCs in radioresistant CRC tissues.

MicroRNAs (miRNAs), a family of small (19-25 nucleotides) endogenous non-coding RNA, negatively regulate at least 30% of human gene expressions at the post-transcriptional level by binding to the target mRNAs often in the 3’-untranslated region (3’UTR) [18–20]. With the understanding of carcinogenesis and tumor progression, it has been recognized that miRNAs play a vital role in regulating cancer stemness, tumor growth, metastasis, and resistance to chemo- and radiotherapy. In CRC, dysregulation of miRNAs including miR-19b, miR-106b and miR-148a has been reported to regulate colorectal CSCs and therapeutic efficacy [21–23]. MiR-7-5p has been proposed as a crucial tumor suppressor involved in regulating cell proliferation and apoptosis, migration and invasion, as well as chemosensitivity and radiosensitivity in various malignant tumors such as breast cancer, non-small cell lung cancer and nasopharyngeal carcinoma [24–27]. However, the role of miR-7-5p in cancer stemness and the response of CRC to radiotherapy has not yet been elucidated.

In our previous study, we initially constructed two radioresistant CRC cell lines, named HCT116-R and RKO-R, using the strategy of fractioned irradiation. RNA-sequencing and qRT-PCR confirmed that miR-7-5p was remarkably decreased both in the radioresistant cell lines, HCT116-R and RKO-R, compared with their parental cell lines, HCT116 and RKO. Meanwhile, miR-7-5p expressed lowly in the CRC tissues recalcitrant to chemo- and radio-therapy. A series of in vitro loss- and gain-of-function studies demonstrated that miR-7-5p suppressed cancer cell stemness and enhanced radiosensitivity in CRC by targeting KLF4. In vivo patient-derived xenograft (PDX) models were used to further investigate the function of nanoparticles packed with miR-7-5p mimics in the response of tumors to radiotherapy. Taken together, these findings revealed the novel roles of miR-7-5p in CRC, and suggested that miR-7-5p could be a potential candidate for combination radiotherapy of human CRC.

## Results

### Lower miR-7-5p expression correlates with radioresistance and poor prognosis in CRC patients

**Fig. 1:**
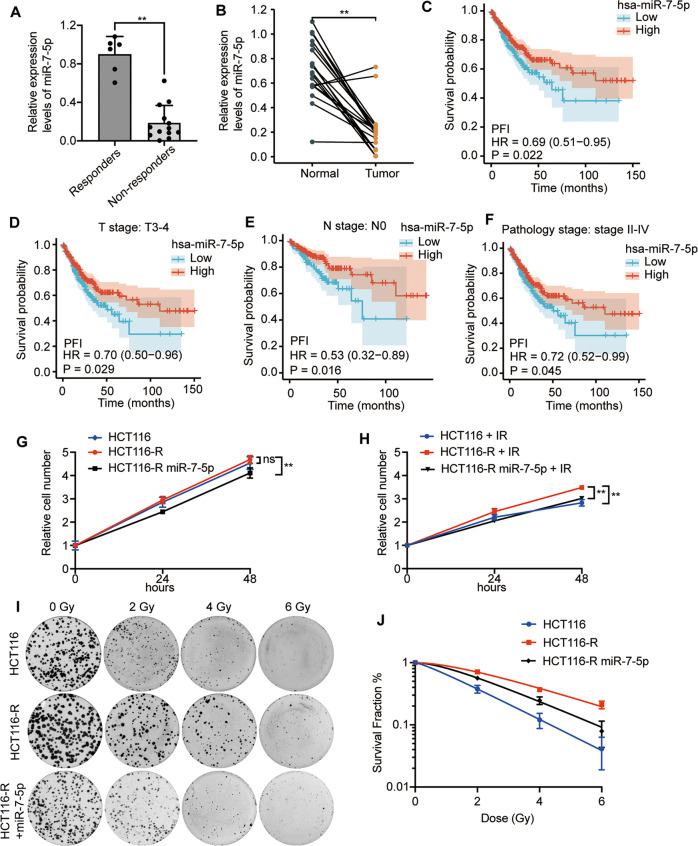
The correlation between the miR-7-5p level and response to radiotherapy in CRC cells. **A** QRT-PCR analysis of miR-7-5p expression in radiosensitive (responders) and radioresistant (non-responders) rectal cancer tumor tissues. **B** QRT-PCR analysis of miR-7-5p expression in CRC tumor tissues and corresponding adjacent normal tissues from 20 CRC patients. Analysis of RNA-seq data retrieved from TCGA-COADREAD illustrated that low expression of miR-7-5p was correlated with shorter progression-free intervals (PFI) **(C)**, and the subsequent subgroup analysis showed that those CRC patients with lower miR-7-5p expression at clinical T3-4 stage (**D**), N0 stage **(E)** or Pathology stage II-IV **(F)** had shorter PFI. **G**, **H** Cell proliferation assays in HCT116, HCT116-R and miR-7-5p-overexpressing HCT116-R cells w/wo 4 Gy irradiation treatment. **I** Representative images of colony formation assays in HCT116, HCT116-R and miR-7-5p-overexpressing HCT116-R cells treated with various doses of irradiation (0, 2, 4, and 6 Gy). **J** The survival analysis in HCT116, HCT116-R and miR-7-5p-overexpressing HCT116-R cells treated with 0, 2, 4 or 6 Gy irradiation, respectively. Data are presented as mean ± SD; qRT-PCR, quantitative real-time PCR; w/wo, with or without; IR irradiation, ns no significant difference; ^**^P < 0.01.

In order to identify the key regulators involving in the response to radiotherapy, two acquired radioresistant CRC cell lines, HCT116-R and RKO-R, were established in our lab as previous described [28]. Of note, miR-7-5p expression was demonstrated to be decreased in HCT116-R and RKO-R in our previous published miRNA sequencing data (GSE159528) compared with their parental HCT116 and RKO cells, respectively [28], which were further validated by qRT-PCR (Supplementary Fig. S2A). Furtherly, to investigate the relationship between miR-7-5p and radiosensitivity of CRC tumor tissues, qRT-PCR assays were performed using 19 pretreatment biopsies to test the differential expression level of miR-7-5p from responders (TRG 0-1) to non-responders (TRG 2-3). The results showed that tissues from patients with poor response to chemoradiation therapy (TRG 2-3) had a significantly lower miR-7-5p expression than those from patients with good response (TRG 0-1) (Fig. 1A). In addition, to demonstrate the clinical relevance of miR-7-5p in CRC, we analyzed the expression of miR-7-5p in our CRC cohort (*n* = 20) and RNA-seq data from TCGA-COADREAD datasets (*n* = 630) retrieved from the TCGA database. The results revealed that miR-7-5p had a significantly lower expression level in 20 CRC tumor tissues compared to their corresponding adjacent normal tissues (Fig. 1B), and patients with lower expression of miR-7-5p had significant shorter progression-free intervals (PFI) (Fig. 1C). Subsequently, subgroup analysis was performed, which indicated that patients with lower miR-7-5p expression at clinical T3-4 stage, N0 stage or Pathology stage II-IV also had significant shorter PFI (Fig. 1D–F). Taken together, these results provided evidence that low miR-7-5p expression correlated with poor response to nCRT and unfavorable prognosis in a clinical setting in CRC, which thus could be potentially served as a candidate biomarker and a therapeutic target for CRC.

### MiR-7-5p promotes radiosensitivity of CRC cell lines

To further explore the biology of miR-7-5p in CRC, we evaluated the effects of miR-7-5p on radiosensitivity of CRC cell lines by using in vitro models. The CCK-8 analyses indicated that HCT116-R grew faster than its parental cells (HCT116) when exposed to 4 Gy within 48 h, but not under normal conditions. Meanwhile, the cell proliferation was obviously decreased in miR-7-5p-overexpressing HCT116-R and RKO-R cells compared to the control cells (HCT116-R and RKO-R) w/wo (with or without) irradiation (Fig. 1G, H,Supplementary Fig. S4B). Conversely, suppression of miR-7-5p could significantly enhance cell proliferation in HCT116 cells under 4 Gy radiation (Supplementary Fig. S3A) and in RKO cells w/wo irradiation (Supplementary Fig. S4A). Furtherly, the colony-formation assay was carried out following irradiation with different doses of X-ray (0, 2, 4 and 6 Gy). The higher survival rate of HCT116-R compared to HCT116 under irradiation was confirmed again, and overexpression of miR-7-5p could partially relieved the elevated colony formation ability in radioresistant HCT116-R cells (Fig. 1I, J). As demonstrated in Fig. 1J and Table 1, the cell survival curves, fitting the data into a single-hit, multi-target model, revealed that HCT116-R exhibited increased survival fraction and greater values of SF2, D0, Dq, and N compared with its parental HCT116 cells treated with various doses of irradiation. However, compared to HCT116-R cells, the survival fraction and the values of SF2, D0, Dq and N cells significantly decreased in HCT116-R cells treated with miR-7-5p mimics plus irradiation; the sensitivity enhancement ratio (SER) was 1.43. Taken together, the results of CCK-8 assay and colony-formation assay confirmed that miR-7-5p could suppress CRC cell proliferation and enhance the sensitivity of CRC cells to radiotherapy.

**Table 1:** The radiosensitive parameters of HCT116, HCT116-R and miR-7-5p-overexpressing HCT116-R cells.

Parameter	HCT116	HCT116-R	HCT116-R miR-7-5p
SF2	0.38	0.71	0.56
D0	1.73	2.72	2.03
Dq	0.40	1.70	1.18
N	1.26	1.87	1.79
SER			1.43

*SF2* surviving fraction at 2 Gy, *D0* mean lethal dose, *Dq* quasi-threshold dose, *N* extrapolation number, *SER* sensitization enhancement ratio. SER = Dq in HCT116-R group/Dq in HCT116-R miR-7-5p group. SER > 1 indicates radiosensitization.

### MiR-7-5p enhances radiation-induced apoptosis of CRC cell lines

**Fig. 2:**
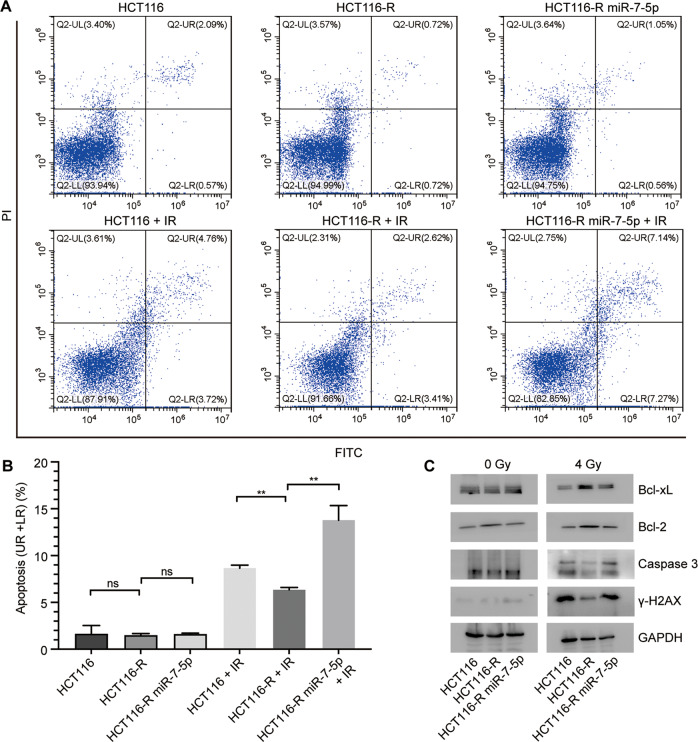
MiR-7-5p promoted the irradiation-induced apoptosis in CRC cells. **A** Representative images of apoptosis in HCT116, HCT116-R and miR-7-5p-overexpressing HCT116-R cells in 48 h after treatment with 0 or 4 Gy irradiation. **B** The early and late apoptotic cell rates (UR + LR) were quantified. **C** Western blot assays showed the expression changes of apoptosis-related genes, including Bcl-xL, Bcl-2 and caspase 3, and a DNA-damage marker γ-H2AX in HCT116, HCT116-R and miR-7-5p-overexpressing HCT116-R cells after 0 or 4 Gy irradiation treatment. UL upper left quadrant, UR upper right quadrant, LL lower left quadrant, LR lower right quadrant, Data are presented as mean ± SD, IR irradiation (4 Gy); ns no significant difference,^**^P < 0.01.

In general, ionizing radiation (IR) could induce DNA damage in cells, and subsequently trigger pro-apoptotic signaling. In order to detect the effect of miR-7-5p on apoptosis of CRC cells w/wo irradiation, we carried out Annexin V-FITC/PI staining and flow cytometry analysis. As expected, HCT116-R cells were refractory to apoptosis induced by irradiation (4 Gy) compared with its parental HCT116 cells, while the apoptotic rate of HCT116-R did not differ from that of HCT116 under normal conditions. Overexpression of miR-7-5p could considerably enhance the IR-induced apoptosis of HCT116-R from 6.33 ± 0.27% to 13.77 ± 1.57% after irradiation of 4 Gy doses, but not under normal conditions (Fig. 2A, B). Furtherly, western blot analysis was performed (Fig. 2C), illustrating that there was no significant difference in the expression levels of Bcl-xL, Bcl-2, caspase 3 and γ-H2AX among the three groups (HCT116, HCT116-R and HCT116-R miR-7-5p) treated without irradiation. However, after 4 Gy irradiation, the expression of anti-apoptotic proteins Bcl-xL and Bcl-2 increased and the expression of pro-apoptotic protein caspase 3 and the DNA damage marker γ-H2AX decreased in HCT116-R compared with its parental cells (HCT116). The tendencies of relevant proteins were notably reversed by overexpressing miR-7-5p in HCT116-R cells. Consistent with these findings, miR-7-5p knockdown in HCT116 significantly inhibited the early apoptotic cell proportion of HCT116 cells at 48 h after 4 Gy X-ray irradiation (Supplementary Fig. S3B, C), and induced the expression of anti-apoptotic proteins Bcl-xL and Bcl-2, and decreased the expression of pro-apoptotic protein caspase 3 and the DNA damage marker γ-H2AX (Supplementary Fig. S3D). In addition, overexpression of miR-7-5p in RKO-R cells could promoted apoptosis, while downregulating miR-7-5p inhibited apoptosis of RKO cells w/wo 4 Gy irradiation (Supplementary Fig. S4 C–F). Taken together, these observations suggested that miR-7-5p restoration could sensitize cells to IR-induced apoptosis in CRC cells and inhibit the repair of IR-induced DNA damage.

### MiR-7-5p suppresses cancer cell stemness in CRC with or without IR

**Fig. 3:**
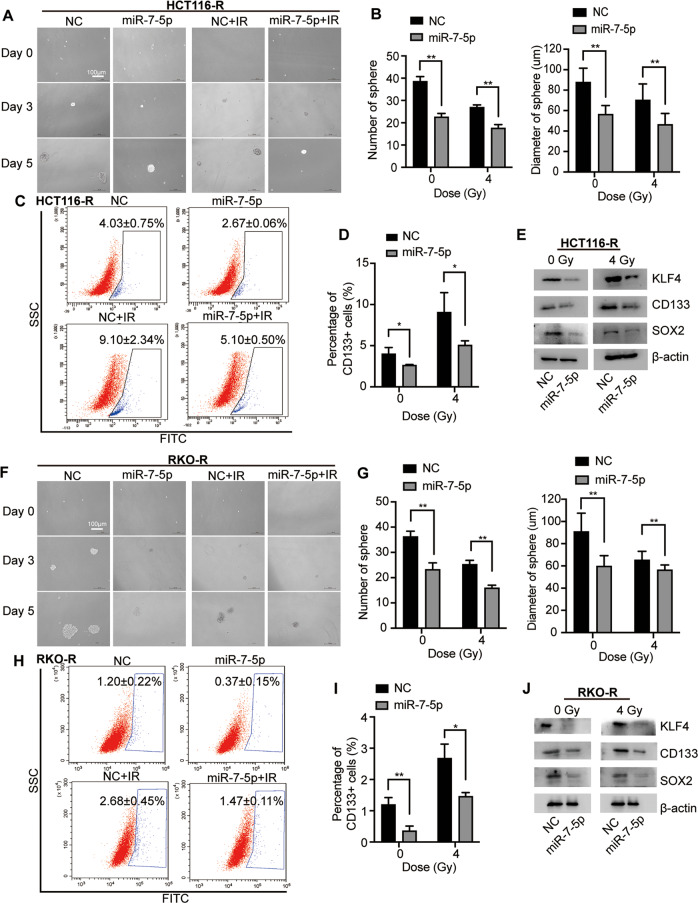
MiR-7-5p suppressed cancer cell stemness in CRC cells. **A** Sphere formation assays using HCT116-R cells after overexpression of miR-7-5p w/wo 4 Gy irradiation treatment. **B** Quantitative analysis of **A** showed that overexpression of miR-7-5p reduced the sphere number and diameter in HCT116-R cells w/wo 4 Gy irradiation treatment. **C** miR-7-5p overexpression in HCT116-R decreased the CD133^+^ CSC subpopulation under irradiation of 0 or 4 Gy. **D** Quantitative analysis of **C**. **E** Gene expression analyses showed negative regulation of stemness genes including KLF4, CD133 and SOX2 at the protein levels by miR-7-5p overexpression in HCT116-R cells. **F** Sphere formation assays using RKO-R cells after overexpression of miR-7-5p w/wo 4 Gy irradiation treatment. **G** Quantitative analysis of **(F)** showed that overexpression of miR-7-5p decreased the sphere number and diameter in RKO-R cells. **H** miR-7-5p overexpression in RKO-R cells decreased the CD133^+^ CSC subpopulation under irradiation of 0 and 4 Gy. **I** Quantitative analysis of **H. J.** Gene expression analyses showed negative regulation of stemness genes including KLF4, CD133 and SOX2 at the protein levels by miR-7-5p overexpression in RKO-R cells. Data are presented as mean ± SD; w/wo, with or without; IR, irradiation (4 Gy); ^*^P < 0.05; ^**^P < 0.01.

**Fig. 4:**
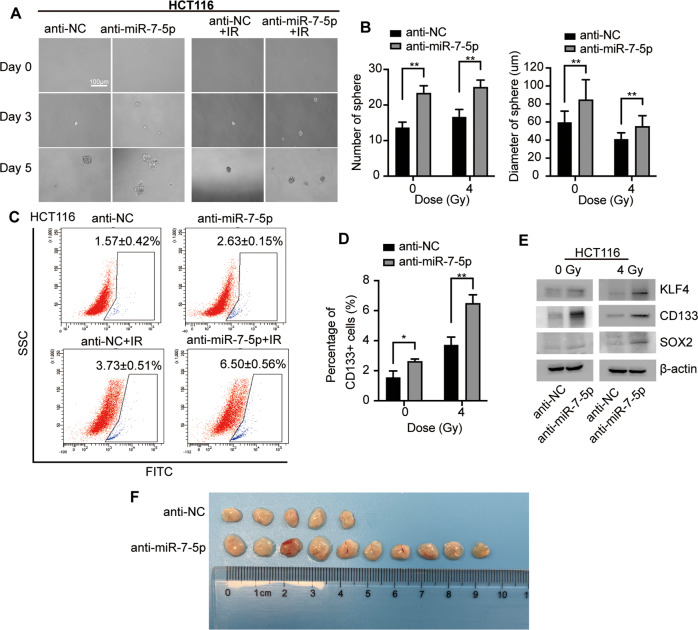
Knockdown of miR-7-5p induced cancer cell stemness of HCT116 with or without irradiation. **A** Sphere formation assays using HCT116 cells after knockdown of miR-7-5p w/wo 4 Gy irradiation. **B** Quantitative analysis of **A** showed that downregulated miR-7-5p increased the sphere number and diameter in HCT116 treated with 0 or 4 Gy irradiation. **C** miR-7-5p knockdown in HCT116 increased the CD133^+^ CSC subpopulation under irradiation of 0 or 4 Gy. **D** Quantitative analysis of **C**. **E** Gene expression analyses showed positive regulation of stemness genes including KLF4, CD133 and SOX2 at the protein levels by knockdown of miR-7-5p in HCT116. **F** In vivo tumor initiation experiments illustrated that 5/10 (anti-NC group) versus 10/10 (anti-miR-7-5p group) of the injected HCT116 cells grew tumors eventually. Data are presented as mean ± SD; IR, irradiation (4 Gy); ^*^P < 0.05; ^**^P < 0.01.

Accumulating evidences have indicated that colorectal CSCs contributes to tumorigenesis and increased resistance to various therapeutic approaches, including chemo- and radiotherapy [15, 29]. Sphere formation assays were performed identifying that overexpressing miR-7-5p inhibited the CRC cell stemness by decreasing both sphere number and sphere size w/wo 4 Gy irradiation (Fig. 3A, B). The changes of the CD133^+^ CSC subpopulation were further detected by flow cytometry analysis (Fig. 3C, D), indicating the proportion of CD133^+^ cells decreasing from 4.03 ± 0.75% to 2.67 ± 0.06% without irradiation, and reducing from 9.10 ± 2.34% to 5.10 ± 0.50% with 4 Gy irradiation in HCT116-R following miR-7-5p overexpression. Moreover, to clarify the underlying mechanisms governing the regulation of CSC by miR-7-5p in CRC cells, a group of stemness-related proteins were detected by western blot assay, illustrating that overexpressing miR-7-5p remarkably decreased the expression of KLF4, CD133 and SOX2 w/wo irradiation (0 or 4 Gy; Fig. 3E). Similar results were obtained from RKO-R (Fig. 3F–J). Consistent with these findings, miR-7-5p knockdown in HCT116 significantly enhanced the sphere formation capability (Fig. 4A, B) and promoted the percentage of CD133^+^ CSC subpopulation (Fig. 4C, D) with 0 or 4 Gy irradiation. The stemness genes were upregulated by miR-7-5p-knockdown in HCT116 after irradiation with 0 or 4 Gy (Fig. 4E). Moreover, in vivo tumor initiation experiments were performed, and the results showed that 5/10 (anti-NC group) versus 10/10 (anti-miR-7-5p group) of the injected HCT116 cells grew tumors eventually (Fig. 4F). Overall, these results demonstrated that CRC cells with lower expression level of miR-7-5p have acquired an increase in cancer stemness under normal and irradiation conditions, which may account for the potential inhibitory effect of miR-7-5p on CRC cell proliferation and resistance to radiotherapy.

### KLF4 is a direct target gene of miR-7-5p in CRC cells

**Fig. 5:**
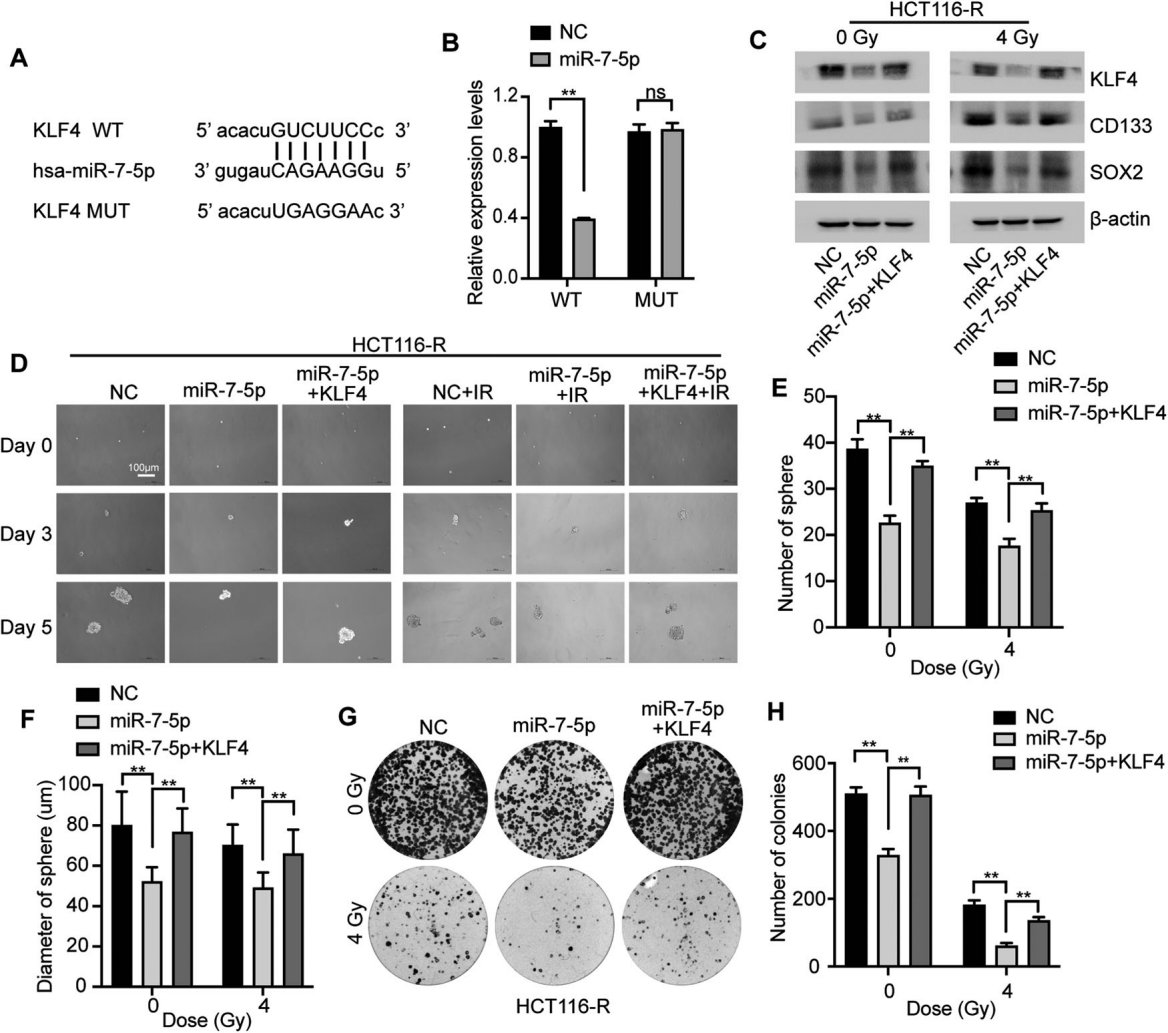
MiR-7-5p suppressed the cancer cell stemness and conferred radiosensitivity in CRC cells by directly targeting KLF4. **A** Wild type (WT) and mutant (MUT) 3’-UTR binding sites in KLF4 for miR-7-5p. **B** The relative luciferase activity was measured in 293 T cells after co-transfecting either WT or MUT pGL3-reporter luciferase vector with miR-7-5p mimics (miR-7-5p) or negative control (NC). **C** Adding back of KLF4 rescued the miR-7-5p-suppressed stem gene expression including CD133 and SOX2 in HCT116-R cells w/wo 4 Gy irradiation treatment. **D** Sphere formation assays using HCT116-R cells after transfection with miR-7-5p mimics w/wo KLF4 under 0 or 4 Gy irradiation. **E, F** Quantitative analysis of **D** revealed that adding back of KLF4 rescued the miR-7-5p-suppressed sphere formation. **G** Colony formation assays of HCT116-R cells after transfection with miR-7-5p mimics w/wo KLF4 under 0 or 4 Gy irradiation treatment. **H** Quantitative analysis of **G** demonstrated that adding back of KLF4 rescued the miR-7-5p-suppressed colony formation ability under irradiation of 0 or 4 Gy. Data are presented as mean ± SD; w/wo, with or without; IR, irradiation (4 Gy); ns, no significant difference;^**^P < 0.01.

Computer-aided bioinformatics analysis with TargetScan software (http://www.targetscan.org) was performed to identify the potential target genes of miR-7-5p. The results showed that the 3’-UTR of human KLF4, a cancer stemness-related gene, was predicted to contain a binding site for the seed sequence of miR-7-5p (Fig. 5A). Western blot analysis has revealed that miR-7-5p could negatively modulate the expression of KLF4 (Fig. 3E–J, and Fig. 4E). To further confirm whether KLF4 is a direct target of miR-7-5p, dual-luciferase reporter assay was performed uncovering that the luciferase activity in 293 T cells was strikingly decreased when co-transfected with WT-KLF4-3’-UTR and miR-7-5p compared with controls, while the cells co-transfected with MUT-KLF4-3’-UTR and miR-7-5p showed unaltered luciferase activity (Fig. 5A, B). The observation above validated that miR-7-5p suppressed the expression of KLF4 by directly binding to its 3’-UTR.

### Restoration of KLF4 expression partially rescues the enhanced radiosensitivity and decreased cancer stemness of CRC cells mediated by miR-7-5p

To further determine whether miR-7-5p enhanced radiosensitivity and decreased cancer stemness of CRC cells by targeting KLF4, rescue experiments were performed in HCT116-R cells. Western blotting assays confirmed the restoration of KLF4 expression by transfecting the pcDNA3.1/KLF4 vector in the miR-7-5p-overexpression HCT116-R cells with 0 or 4 Gy irradiation (Fig. 5C), accompanied by the re-acquisition of the expression of stemness-related genes, CD133 and SOX2. Moreover, sphere formation assays revealed that co-transfected with miR-7-5p and pcDNA3.1/KLF4 abolished the miR-7-5p-induced suppression of the tumor sphere-forming capacity measured by quantifying the number and diameter of spheres, and the same trend occurred in the presence of irradiation at a dose of 4 Gy (Fig. 5D–F). In addition, transfected cells were irradiated with 0 or 4 Gy X-rays for detecting the colony formation abilities. Colony formation assays elucidated that ectopic expression of KLF4 by pcDNA3.1/KLF4 significantly relieved the reduced cell proliferation capacity triggered by miR-7-5p in HCT116-R cells w/wo irradiation (Fig. 5G, H). Taken together, these results revealed that miR-7-5p decreased the cancer stemness and enhanced radiosensitivity of CRC cells by targeting KLF4.

### Therapeutic delivery of tumor-targeted nanoparticles carrying miR-7-5p mimics enhances radiosensitivity in PDX models of CRC

**Fig. 6:**
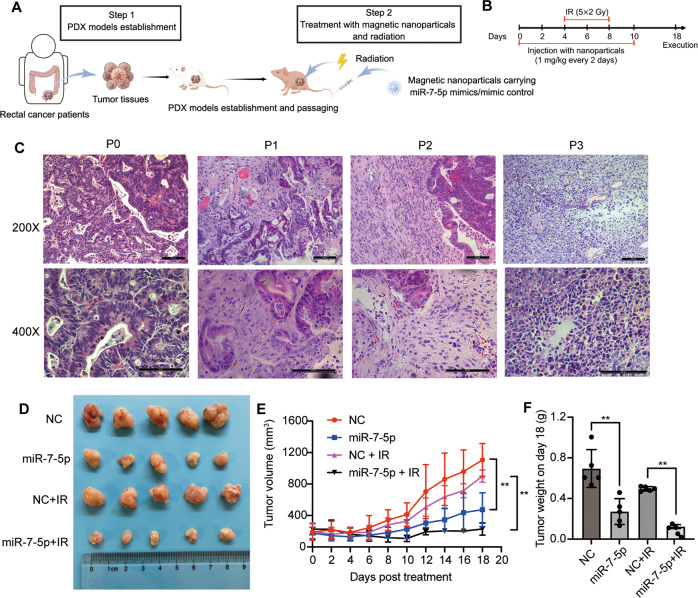
Therapeutic effect of tumor-targeted delivery of nanoparticles carrying miR-7-5p into the CRC PDX mice by enhancing radiosensitivity. **A, B** Schematic representation of the procedure for the PDX establishment and tail vein injection of Zn0.4Fe2.6O4@SiO2 magnetic nanoparticles carrying either miR-7-5p mimics or negative control w/wo radiation therapy. **C** The H&E staining of passage 0, 1, 2 and 3 (P0-3) of PDX-derived tumor tissues (Magnification, 200× and 400×; bar = 100 μm). **D** Tumor images isolated from the mice in the 4 groups: negative control (NC), miR-7-5p, NC plus irrradiation (NC + IR) and miR-7-5p plus irradiation (miR-7-5p + IR). **E** The tumor growth curves of the mice. **F** The weight of tumors in mice. Data are presented as mean ± SD; w/wo, with or without; IR irradiation; ^**^P < 0.01.

In order to examine the therapeutic potential of miR-7-5p to overcome radioresistance in CRC treatment in vivo, the PDX models were established with tumors derived from a rectal cancer patient who presented with radiotherapy-resistance. The contrast-enhanced pelvic MRI images of the patient before and after nCRT were shown in Supplementary Fig. S1. The schematic representation of the procedure (Fig. 6A) was drawn by Figdraw (www.figdraw.com). The PDX mice (P3) were administrated with magnetic HA-nanoparticles carrying miR-7-5p mimic oligonucleotides through tail vein injection in combination with the xenografted tumors exposure to fractioned X-ray irradiation **(**Fig. 6A, B). Histologically, tumors at passage 0 (P0) were moderately differentiated adenocarcinomas exhibiting glandular growth patterns, while tumors that had been passaged 3 times (P3) were poorly-differentiated adenocarcinomas (Fig. 6C). The change in malignant pathological type may be attributable to clonal expansion of cells with survival advantages. When the average tumor volume reached 200 mm^3^, the mice were randomly divided to 4 groups: negative control (NC), miR-7-5p, NC plus irradiation (NC + IR) and miR-7-5p plus radiation (miR-7-5p + IR). The combination of miR-7-5p mimic oligonucleotides delivered by magnetic HA-nanoparticles and radiotherapy significantly reduced the tumor size and delayed the tumor growth (Fig. 6D, E), which was further validated with the tumor weight (Fig. 6F). These results reinforced the therapeutic potential of miR-7-5p to serve as a radiosensitizer to improve the prognosis of CRC.

## Discussion

For optimization of the therapeutic efficacy, radiotherapy is recommended as a neoadjuvant treatment for patients with clinical stage T3 or T4 and/or node-positive tumors, aiming to improve the sphincter-preserving rate, enhance the local control and reduce the risk of local recurrence [4]. In clinical practice, tumor regression after preoperative radiotherapy shows marked variability. Unfortunately, the molecular mechanisms underlying resistance to radiotherapy in colorectal cancer are still poorly recognized.

Recently, increasing attention is being paid to the regulatory functions of miRNAs in cancer therapy resistance, especially radiotherapy [30, 31]. Radiation conditions can induce dysregulation of miRNAs, and some miRNAs regulate tumor radiosensitivity involved in diverse aspects of cellular and biological processes by interacting with the target genes and vital signaling pathways. It was reported that miR-200c enhances radiosensitivity by inducing G2/M and sub-G1 phase arrest in esophageal cancer [32]. MiR-191 promotes resistance of prostate cancer to radiation by targeting retinoid X receptor alpha (RXRA) [33]. MiR-29b increases radiosensitivity by modulating the oxidative stress response and inhibiting repair of DNA double-strand break (DSB) [34]. In our previous study, we initially established two radioresistant CRC cell lines, HCT116-R and RKO-R, with repeated X-ray radiotherapy [28]. RNA-seq analysis and qRT-PCR results confirmed that miR-7-5p was significantly downregulated in radioresistant cells and rectal cancer tissues, suggesting its potential effect on regulating the radiosensitivity of these cells.

MiR-7-5p, a mature miRNA, which is derived from the 5’ arm of three microRNA precursors in human genome, miR-7-1, miR-7-2 and miR-7-3. MiR-7-5p has been verified to be expressed at low levels and act as a tumor suppressor due to its role in suppressing tumor growth, invasion and metastasis in various human malignancies, including breast cancer [24, 35], lung cancer [25], hepatocellular carcinoma [36], etc. Several studies have reported that miR-7-5p regulates the response to radiotherapy, chemotherapy and targeted therapy [26, 37]. Tomita et al. suggested that miR-7-5p is involved in radioresistance via ROS generation in radioresistant HeLa and SAS cells [38]. Lai et al. revealed that miR-7-5p suppresses resistance to doxorubicin through inhibiting DNA homologous recombination repair in small cell lung cancer [26]. Kabir et al. reported that miR-7 can overcome sorafenib-resistance in human hepatocellular carcinoma by suppressing Tyro3 function [37]. However, there is a lack of research with respect to the regulatory role of miR-7-5p in radioresistance of CRC. The aim of this work was to explore the relationship between miR-7-5p and CRC radioresistance as well as the potential fundamental mechanism(s) in CRC cells.

In our present study, we found that overexpression of miR-7-5p had a significantly inhibitory effect on the proliferation and clonogenic cell survival under both normal and radiation conditions. These findings were consistent with a previous study, which revealed that miR-7-5p can be served as a tumor suppressor to inhibit CRC proliferation and migration [39]. In addition, Ionizing radiation could cause irreparable DNA damage, which subsequently leads to cell apoptosis and tumor death [40]. The flow cytometry analysis demonstrated that miR-7-5p overexpression markedly increased the apoptosis rate, and the effect was more pronounced when combined with irradiation of 4 Gy. These results were supported with the expression levels of apoptosis-related proteins (Bcl-2, Bcl-xL, Caspase 3). The accumulation of γ-H2AX occurred in CRC cells after overexpressing miR-7-5p under radiation conditions. Taken together, the gain- and loss-of-function studies elucidated that ectopic expression of miR-7-5p could enhance radiosensitivity of CRC cells, evidenced by decreased cell proliferation and survival fractions, enhanced IR-induced apoptosis and suppressed DNA damage repair in the presence of IR in vitro.

Our in vitro findings were substantiated by the in vivo studies in PDX models derived from one rectal cancer patient who presented with resistance to neoadjuvant chemo-radiotherapy. The results demonstrated that miR-7-5p overexpression significantly inhibits tumor growth treated w/wo radiotherapy, and thus miR-7-5p improved the sensitivity to radiation of the PDX tumor tissues. Therefore, miR-7-5p is considered as a tumor suppressor and a potential candidate radiosensitizer.

Multiple studies have identified that cancer stem cells exhibit self-renewal ability, efficient DNA damage repair and resistance to irradiation-induced cell apoptosis, which contribute to the failure of radiotherapy to eliminate the tumor and ultimately result in radioresistance [41–43]. In breast cancer cells, miR-142-3p inhibits cancer cell stemness and promotes radiosensitivity [44]. In nasopharyngeal carcinoma cells, miR-124 attenuates cancer stem-like properties and inhibits radioresistance via targeting JAMA [45]. In addition, exosomal miR-19b was reported to increase cancer stemness and induce radioresistance in CRC [21]. In our current study, we identified that miR-7-5p could decrease the proportion of CD133 + cells, attenuate the cell self-renewal capacity and downregulate the expression of cancer stem cell markers in CRC cells treated w/wo radiotherapy. Our findings provided evidence for a direct link between CSCs properties and radiotolerance, and these results suggested that miR-7-5p were involved in the regulation of the response to radiotherapy by inhibiting CSCs self-renewal and maintenance.

KLF4, also known as krüppel-like factor 4, has been widely investigated as a stemness-associated transcription factor, and was confirmed to induce and maintain the self-renewal and pluripotent capacity of stem cells, as well as implicate in cellular reprogramming and cancer initiation [46]. Depending on the cellular context, KLF4 can thus exerts different biological function. A prior study indicated that in response to IR-induced DNA damage, KLF4 exhibits antiapoptotic effect by promoting the expression of p21, leading to cell cycle arrest following DNA repair process, and by suppressing p53-mediated activation of the proapoptotic gene, BAX [47]. In our current study, the results demonstrated that overexpression miR-7-5p downregulated KLF4 in CRC cells leading to the increased apoptotic rates and attenuated radioresistance in the presence of IR. Moreover, overexpression of KLF4 partially rescued IR-induced apoptosis, and increased the radioresistance in miR-7-5p-overexpression HCT116-R cells. This phenomenon indicated that overexpression of miR-7-5p might impair KLF4-mediated anti-apoptosis and DNA damage repair in response to radiation, and miR-7-5p enhanced radiosensitivity by regulating KLF4. In addition, KLF4 is an important stem-cell-inducing factor that help cancer stem cells to acquire and maintain their CSC properties. It is reported that KLF4 is highly expressed in CRC stem cell-enriched spheroid cells and functions as an oncogene for the development of CRC [48]. In our present study, we identified KLF4 as a direct target of miR-7-5p. Restoring of KLF4 in miR-7-5p-overexpressing HCT116-R cells could rescue the decreased cancer stemness and the attenuated radioresistance regulated by miR-7-5p. These data indicated that miR-7-5p functioned as a tumor suppressor by directly targeting KLF4 to suppress cancer stemness, and as a radiosensitizer to inhibit the resistance to radiotherapy in CRC. Moreover, we have previously reported that miR-423-5p could suppress radioresistance by targeting Bcl-xl of CRC cells [28]. In our present study, we found that miR-7-5p could inhibit radioresistance through suppressing the cancer cell stemness by directly targeting KLF4 in CRC cells. Although miR-423-5p and miR-7-5p have similar effects on radiosensitivity in CRC cells, while the two miRNAs exert their effects via the regulation of distinct target genes and different intracellular mechanisms.

In conclusion, our results identified that miR-7-5p was downregulated in radioresistant cells and tissues, and enforced expression of miR-7-5p could increase radiosensitivity by directly targeting KLF4 that inducing impaired cancer stem cell properties in CRC cells. Both in vitro and in vivo experiments suggested the therapeutic potential of the combination radiotherapy with systemic delivery of miR-7-5p in the treatment of CRC.

## Materials and methods

### Patients and tissue samples

Fresh biopsies of rectal cancer tumors were collected from patients treated between January 2020 and September 2021 at Shanghai East hospital and Changhai hospital in Shanghai, China. The patients were diagnosed as locally advanced rectal cancer (LARC) with stage II or III according to the American Joint Committee on Cancer (AJCC) 8th edition, and met the clinical criteria for neoadjuvant chemoradiotherapy (nCRT). The patients received pelvic locoregional radiotherapy (45-50.4 Gy) combined with capecitabine or 5-fluorouracil, which served as a radiation sensitizer. After surgery, the responses to nCRT were assessed by the tumor regression grade (TRG 0-3) according to the American Joint Committee on Cancer (AJCC) criteria [49], which was assigned by two consultant pathologists. Patients were classified into two different response groups: responders (TRG 0-1) and non-responders (TRG 2-3). The CRC tumor tissues and corresponding adjacent normal tissues were collected at Shanghai East hospital between June 2021 and August 2021. After excision, the fresh tissues were immediately immersed in liquid nitrogen. The method of samples collection was simple random sampling.

To establish PDX, a tumor biopsy was obtained before nCRT from a 70-year-old Chinese male diagnosed with rectal cancer (cTxN+M0) at Shanghai East hospital, who had received standard fractioned radiation followed by chemotherapy (50.4 Gy in 28 fractions with capecitabine). 8 weeks after neoadjuvant therapy, no tumor regressed but showed liver metastatic lesion, and the clinical stage (AJCC) was ycT4N + M1. Where “c” stands for clinical staging based on imaging when no surgical specimen was available, and “y” for staging after neoadjuvant treatment. The response to nCRT was assessed as Progressive Disease (PD) based on RECIST 1.1 guidelines [50]. The patient was considered unfit for surgery and the contrast-enhanced pelvic MRI images before and after nCRT were shown in Supplementary Fig. S1.

Written informed consent forms were obtained from all participants. All the procedures were approved by the Institutional Review Board (IRB) and the Medical Ethics Committee of Shanghai East Hospital and Changhai hospital.

### Bioinformatics analysis

The normalized RNA-seq data from level 3 BCGSC miRNA Profiling and the clinical information of CRC patients (*n* = 630) were obtained from The Cancer Genome Atlas database (TCGA-COAD and TCGA-READ) (https://portal.gdc.cancer.gov/). Patients with incomplete clinicopathological information were exclude.

### Cell lines

The human CRC cell lines HCT116 and RKO were previously purchased from American Type Culture Collection (ATCC). The radioresistant cell lines, HCT116-R and RKO-R, were established in our lab as previously described [28]. Parental cells (HCT116 and RKO) and radioresistant cells (HCT116-R and RKO-R) were cultured in high glucose DMEM medium (Gibco, USA) containing 10% fetal bovine serum (FBS, Gibco) and 1% penicillin-streptomycin liquid (Gibco), and incubated at 37 °C in a 5% CO_2_-humidified atmosphere. Cells tested negative for mycoplasma.

### X-irradiation

A 6-megavolt X-ray beam was generated by a clinical linear accelerator (Varian, EDGE, USA) with a radiation dose rate of 600 MU/min in the Department of Radiation Oncology at Shanghai East Hospital (Shanghai, China). The cells were exposed to varied doses of X-ray irradiation (0, 2, 4, 6 Gy), with a treatment field of 40 × 40 cm^2^, a 1.5 cm-thick bolus material placed on the cover, and the source-to-sample distance of 100 cm. For mice, the size of the irradiation field was 0.7 × 0.7 cm to 1.5 × 1.5 cm adjusted according to the actual size of tumor so as to cover the entire tumor foci. The 1.5 cm-thick multiple pieces of wet cotton gauze equivalent of bolus material were used to cover the tumor surface. The distance from the tumor center to irradiation source was 100 cm. The rest of the body was protected outside the irradiation field. Tumor-targeted radiotherapy was given in five fractions of 2 Gy for up to 5 consecutive days.

### Quantitative real time PCR (qRT-PCR)

Total RNA from cells and tissues were extracted with TRIzol reagent (Invitrogen). The miRNeasy Mini Kit (Qiagen, Valencia, CA) was used to isolate miRNAs. Total RNA was reversely transcribed into cDNA using the PrimeScript™ RT reagent kit (TaKaRa, Otsu, Japan), while miRNA was reversely transcribed using One Step PrimeScript miRNA cDNA Synthesis Kit (Takara). SYBR Premix Ex Taq™ kit (Takara) was used for qPCR analysis in Applied Biosystems QuantStudio 6 (Applied Biosystem, Thermo Fisher Scientific). U6 was chosen as endogenous control for miRNA. The 2^−ΔΔCt^ method was used to analyze the expression data. Primer sequences were as follows: hsa-miR-7-5p, 5′ CGGAAGACTAGTGATTTTGTTG 3′; U6, 5′ GCTCGCTTCGGCAGCACATAT 3′; UR2, 5′ CTAGATCAGCTGGGCCAAGA 3′.

### Western blotting assay

A total of 40 μg protein lysates collected from lysed cells in RIPA Buffer (Beyotime, Shanghai, China) were fractionated on a 10% SDS polyacrylamide gel, and then transferred onto PVDF membrane (Millipore, Temecula, CA, USA). The membranes were blocked in 5% skim milk/TBST for 1 h, and then probed with the primary antibodies to detect the following proteins: KLF4 (ARG 55811, Arigo, Taiwan, China), SOX2 (3579, Cell Signaling Technology, MA, USA), CD133 (ab216323, abcam, Cambridge, United Kingdom), Bcl-2 (sc-492, Santa Cruz biotechnology, CA, USA), Bcl-xL (ab32370, abcam), caspase 3 (ARG54938, Arigo), γH2AX (ab81299, abcam), β-actin (sc-47778, Santa Cruz biotechnology), Tublin (ab7291, abcam) and GAPDH (sc-47724, Santa Cruz). Horseradish peroxidase (HRP)-linked anti-mouse or anti-rabbit IgG served as the secondary antibodies. The full length uncropped original western blots were shown in supplementary materials.

### Oligonucleotide, plasmid construction and transfection

Cells were cultured in 6-well plates transfected with miR-7-5p mimics (miR10000252-1-5, RiboBio, Guangzhou, China), mimic negative control (MIMAT0000295, RiboBio), miR-7-5p inhibitors (miR20000252-1-5, RiboBio) and inhibitor negative control (MIMAT0000295, RiboBio). Transfection efficiency was evaluated by qRT-PCR in HCT116, RKO, HCT116-R and RKO-R cells (Supplementary Fig. S2). For KLF4 overexpression, the full-length KLF4 sequence was amplified and subcloned into a pcDNA-3.1 plasmid (Genomeditech, Shanghai, China). The CRC cells were transiently transfected in serum-free OPTI-MEM (Invitrogen, Carlsbad, CA) using Lipofectamine^TM^ 2000 reagent (Invitrogen) following the instructions from the manufacturer.

### Cell proliferation assay

Transfected HCT116, RKO, HCT116-R and RKO-R cells (2000 cells per well) were plated in 96-well plates. After 24 h incubation, these cells were exposed to 4 Gy X-ray irradiation. After 24 more hours post-irradiation, each well of the plates were added with 10 μl CCK-8 solution (Cell Counting Kit-8; Cellor Lab, China) and incubated for 3 h at 37 °C. A microplate reader determined the optical density values (OD values) at 450 nm.

### Colony formation assay

Transfected cells were seeded at 500 cells/well in a 12-well plate in triplicate. After 24 h, cells were exposed to varied doses of irradiation (0, 2, 4, and 6 Gy). After 7–14 days of incubation, we fixed and stained these cells with crystal violet (Sigma, CA, USA), and the colonies (≥50 cells) were counted. The following formulas were used: plating efficiency (PE) = (number of colonies counted/number of cells plated) × 100%; surviving fraction (SF) = (PE of treated/PE of untreated sample) × 100%. A multi-target single-hit model [28] was used to generate the radiation dose response curves: $SF = 1 - \left( {1 - e^{ - \frac{D}{{D0}}}} \right)^N$. According to the curves, the surviving fraction at 2 Gy (SF2), mean lethal dose (D0), quasi-threshold dose (Dq), extrapolation number (N) and sensitization enhancement ratio (SER) (SER = Dq in the control group/Dq in the experimental group) were calculated. SER > 1 indicates radiosensitization.

### Flow cytometry

Cell apoptosis was detected by flow cytometry analysis using Annexin V-FITC/PI Assay kit (BB-4101, BestBio, Shanghai, China) following the instruction of manufacturer. Briefly, after 48 h post-irradiation (0 or 4 Gy), the cells were digested with trypsin (without EDTA), washed with PBS, and then stained with annexin V-FITC (5 µl) and PI (5 µl). The apoptotic cells were analyzed by flow cytometry (BD Biosciences, San Diego, USA) and Beckman Flow Cytometry Analyzer (Beckman CytoFLEX, CA, USA).

The CD133 expression level was also detected by flow cytometry analysis. The cells were digested with trypsin (without EDTA), and stained with the human anti-CD133/FITC (bs-0395R-FITC, Bioss, Beijing, China). The CD133 expression was analyzed by flow cytometry (BD Biosciences, San Diego, USA) and Beckman Flow Cytometry Analyzer (Beckman CytoFLEX, CA, USA). Each experiment was performed in triplicate.

### Sphere formation assay

After treated with transfection and radiation, the HCT116, HCT116-R and RKO-R cells were seeded into 6-well suspension culture dishes (Corning, United States) at a density of 1000 cells/well. The cells were cultured in DMEM-F12 medium (Gibco) containing human epidermal growth factor (EGF, 20 ng/mL; Sigma), human basic fibroblast growth factor (bFGF, 20 ng/mL; R&D Systems) and B27 supplement (1×; Invitrogen), for 5 days. Spheres more than 40 μm in diameter were counted under a microscope.

### Luciferase reporter assay

The wide or mutated types of KLF4 3′-UTR were cloned into pGL3-reporter luciferase vector (Genomeditech, Shanghai, China) to construct pGL3-KLF4 vectors. After cultured in 24-well plates for 24 h, 293 T cells were co-transfected with either of these pGL3-KLF4 vectors, along with miR-7-5p mimics or negative controls using Lipofectamine^TM^ 2000 reagent (Invitrogen) and cultured for 48 h. Dual-luciferase reporter assays were performed to detect the luciferase activities by using the dual- luciferase reporter assay system (Promega, Madison, WI, USA) according to the manufacturer’s protocol.

### In vivo tumor initiation experiments

Four to six-week-old athymic male Balb-c-nude mice (GemPharmatech, Nanjing, China) were used and 10 mice were randomly divided into two groups by simple random sampling method. The investigator was blinded with respect to the group allocation during experiments. HCT116 cells were transfected with either miR-7-5p inhibitors (anti-miR-7-5p) or inhibitor NC (anti-NC) and were injected subcutaneously into right and left flanks of the mice at 500 cells/site mixed with 40% Matrigel. The number of tumors formed was observed 35 days post injection.

### In vivo patient-derived rectal cancer xenograft model

The PDX models were established by LIDE Biotech (Shanghai, China) using immunodeficient 4 to 6-week-old male NOD-Prkdcem26Cd52Il2rgem26Cd22/Nju (NCG) mice (GemPharmatech). Once established, the PDX mice were maintained by serial passaging in athymic Balb-c-nude mice (GemPharmatech) as previously described [51]. The third passage (P3) of PDX models was used for the subsequent experiments. After tumor implantation, when the average tumor volume reached 200 mm^3^, the mice were separated randomly into 4 groups (5 animals for each group) by simple random sampling method and were subsequently given magnetic nanoparticles pre-loaded with miR-7-5p mimics or mimic-NC oligo (6 doses of 1 mg/kg each given every 2 days), respectively. The investigator was blinded with respect to the group allocation during experiments. The magnetic nanoparticles were previously used in our lab [52], which were prepared with the modified Zn_0.4_ Fe_2.6_ O_4_ @SiO_2_ nanoparticles and miR-7-5p mimic oligo or mimic-NC oligo (2: 1 at room temperature). After injection, a handheld magnet was placed on each tumor site for 1 h. Localized radiation therapy (2 Gy per day for 5 consecutive days, total 10 Gy) was delivered only to tumors after 3 doses of magnetic nanoparticles. The tumors volume was measured every 2 days and calculated as follows: volume = length×width^2^× 0.5. When the tumor volume in the vehicle group reached 1200 mm^3^, tumors of all mice were separated and weighed.

Formal approval to carry out all animal experiments was granted by the Institutional Animal Care and Use Committee (IACUC) and Institutional Ethics Committee at Shanghai LIDE Biotech and Tongji university school of medicine. All animal experiments followed the established national and international ethical regulations for animal research.

### Statistical analysis

Analyses of differences between 2 groups were performed using Student’s *t* test. Statistical analyses of differences among more than 2 groups were demonstrated using one-way ANOVA test. The variance was similar between the groups that are being statistically compared. The survival rates of Progress Free Intervals (PFI) from TCGA-COADREAD were evaluated by Kaplan–Meier (KM) curve with Cox regression analysis. Data were presented with mean±standard deviation (SD). *P* < 0.05 was considered statistically significant, all P values are bilateral/two-sided. The statistical software was the GraphPad Prism version 8 (GraphPad Software, La Jolla, CA, USA).

## Supplementary information

Supplementary Figures

Original Data File

## Data Availability

Data generated and analyzed during this study are included in this published article and its additional files.
