# Supplementary material for: MiR-7-5p/KLF4 signaling inhibits stemness and radioresistance in colorectal cancer
Source: Cell Death Discov. 2023 Feb 2;9:42. doi: 10.1038/s41420-023-01339-8 (PMC9894908; doi:10.1038/s41420-023-01339-8)
Supplement: Supplementary file 1 — Supplementary Figures [file 41420_2023_1339_MOESM1_ESM.docx]

**MiR-7-5p****/KLF4 signaling inhibits stemness and radioresistance in colorectal cancer**

Yuanyuan Shang^1, #^, Zhe Zhu^1, #^, Yuanyuan Zhang^1, #^, Fang Ji^1^, Lian Zhu^2^, Mengcheng Liu^1^, Yewei Deng^1^, Guifen Lv^1^, Dan Li^1^, Bing Lu^1,^ *, Zhuqing Zhou^1,^ *, Chuan-gang Fu^1,^ *

^1^ Department of Colorectal Surgery, Department of General Surgery, Shanghai east Hospital, School of Medicine, Tongji University, Shanghai 200120, China

^2^ Department of Radiation Oncology, Shanghai East Hospital, School of Medicine, Tongji University, Shanghai 200120, China

**Supplementary Figures S1 to S4**

**
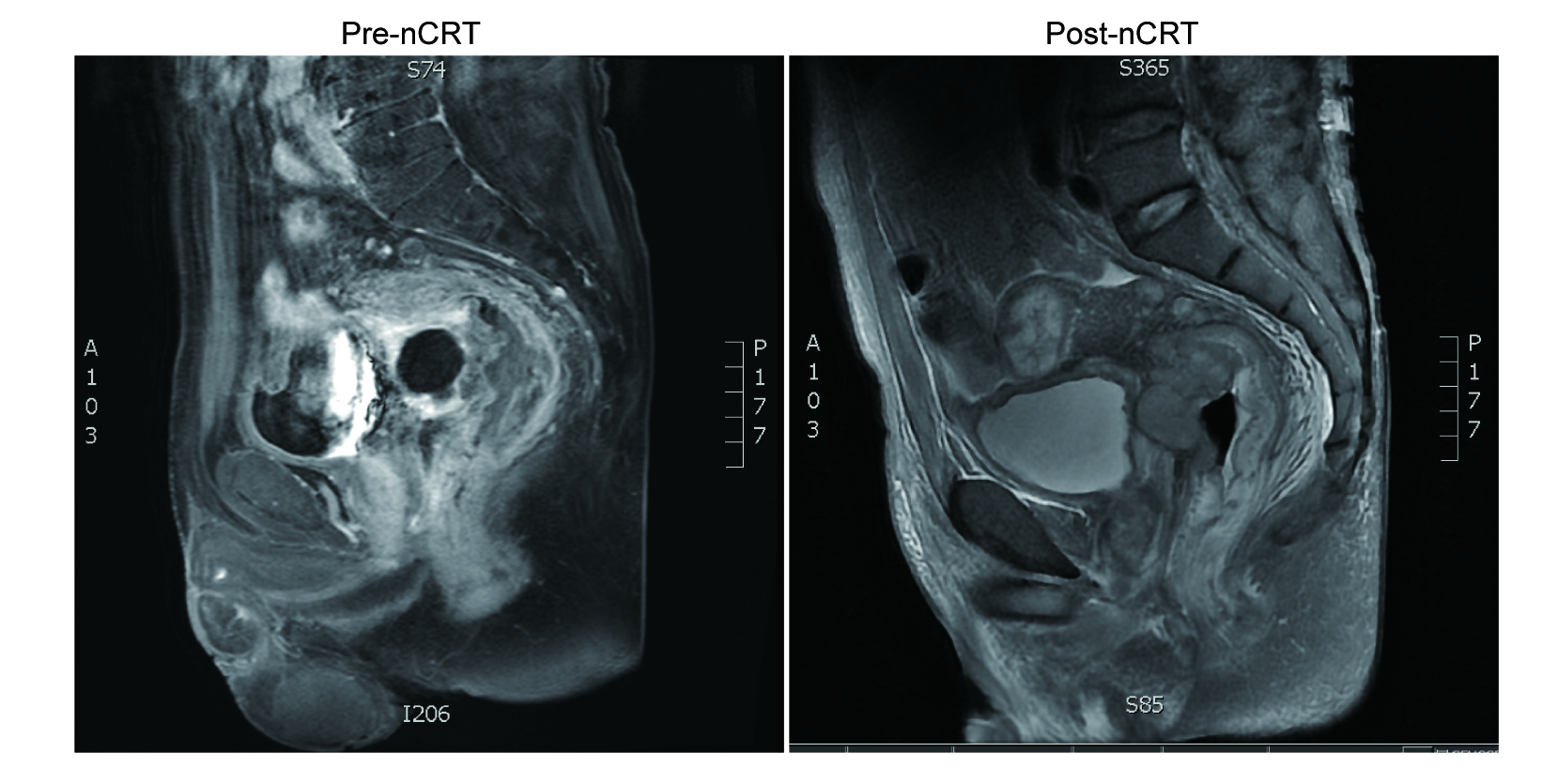
**

**Figure S1. The contrast-enhanced pelvic MRI images before and after neoadjuvant chemoradiotherapy of the patient with locally advanced rectal cancer.** Pre-nCRT, before neoadjuvant chemoradiotherapy, post-nCRT, after neoadjuvant chemoradiotherapy.

**
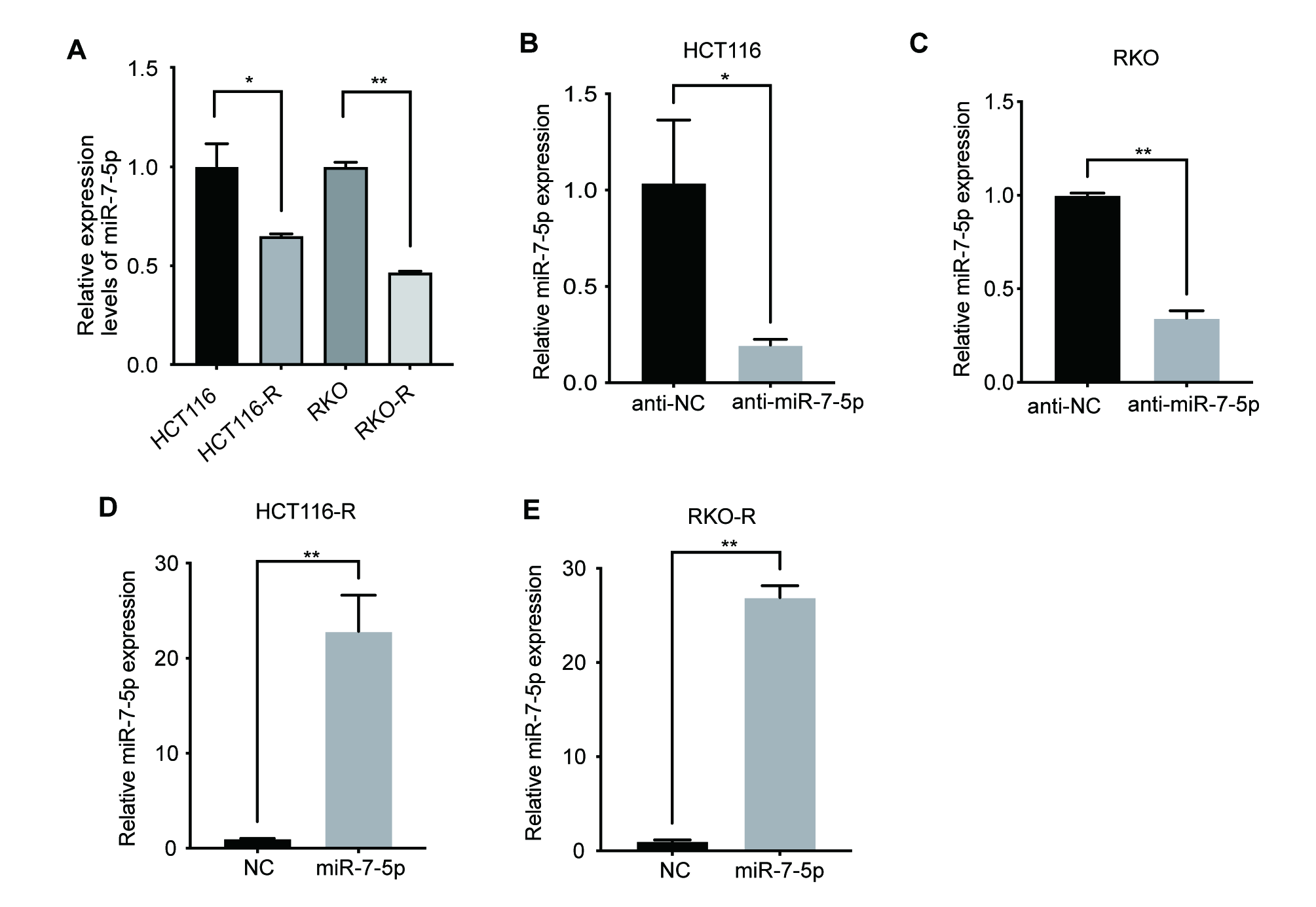
**

**Figure S2. Analysis of transfection efficiency by quantitative real-time PCR. (A)** Quantitative real-time PCR (qRT-PCR) analysis of miR-7-5p expression in radioresistant cells (HCT116-R and RKO-R) and their parental cells (HCT116 and RKO). **(B-C)** Quantitative validation of miR-7-5p knockdown in HCT116 **(B)** and RKO **(C)**. **(D-E)** Quantitative validation of miR-7-5p overexpression in HCT116-R **(D)** and RKO-R **(E)**.

**
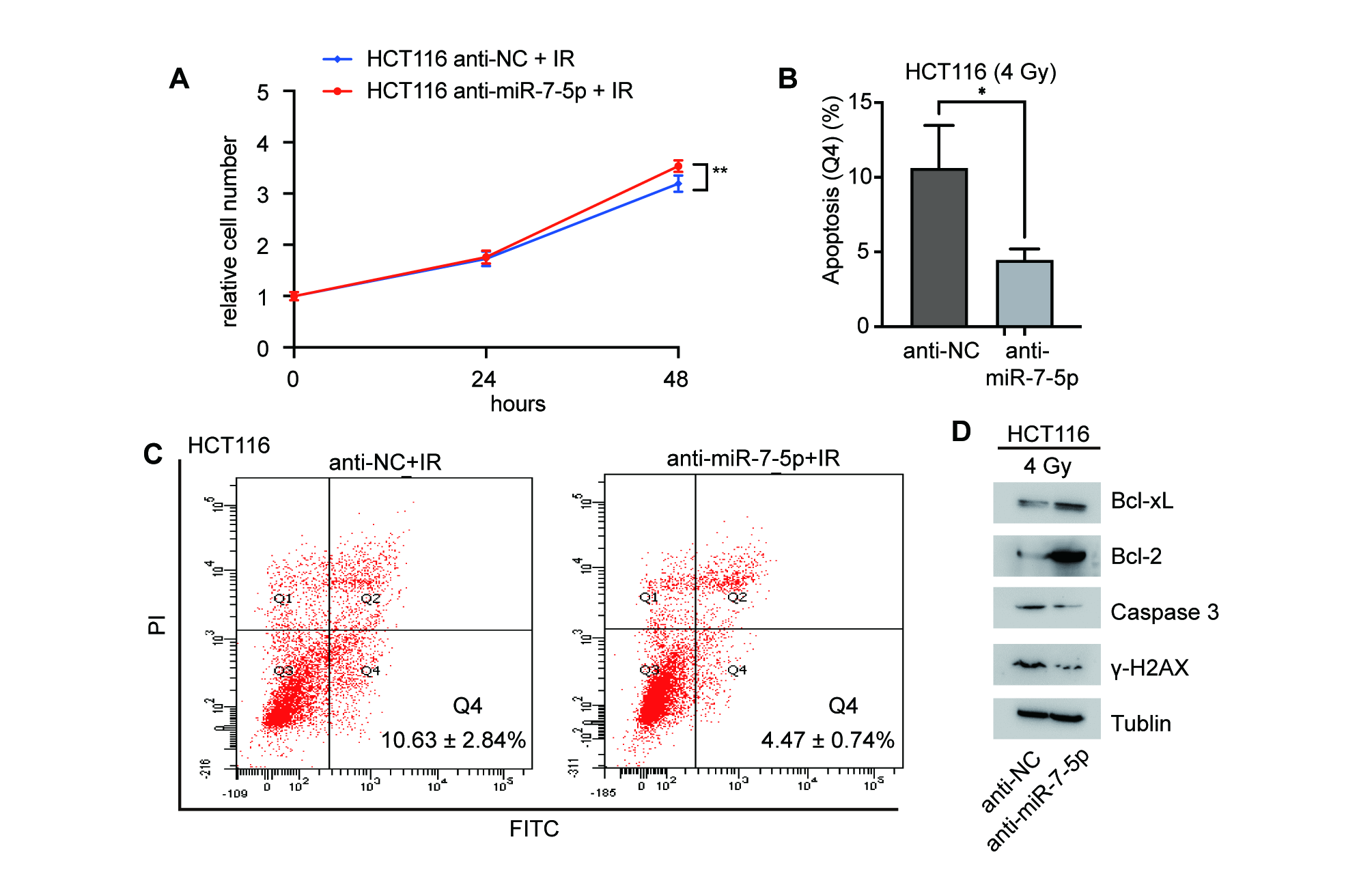
**

**Figure S3. Knockdown of miR-7-5p promoted cell proliferation and inhibited radiation-induced apoptosis of HCT116 under 4 Gy irradiation. (A)** CCK-8 assays illustrated that the cell proliferation increased in HCT116 after knockdown of miR-7-5p with 4 Gy irradiation. **(B-C)** The early apoptosis rates (Q4) decreased in HCT116 after knockdown of miR-7-5p when exposed to 4 Gy irradiation. **(D)** Western blot assay showed the expression changes of apoptosis-related molecules, including Bcl-xL, Bcl-2 and Caspase 3, and a DNA damage marker γ-H2AX in HCT116 after miR-7-5p knockdown under 4 Gy irradiation. Data are presented as mean ± SD; IR, irradiation (4 Gy); *P < 0.05; **P < 0.01.


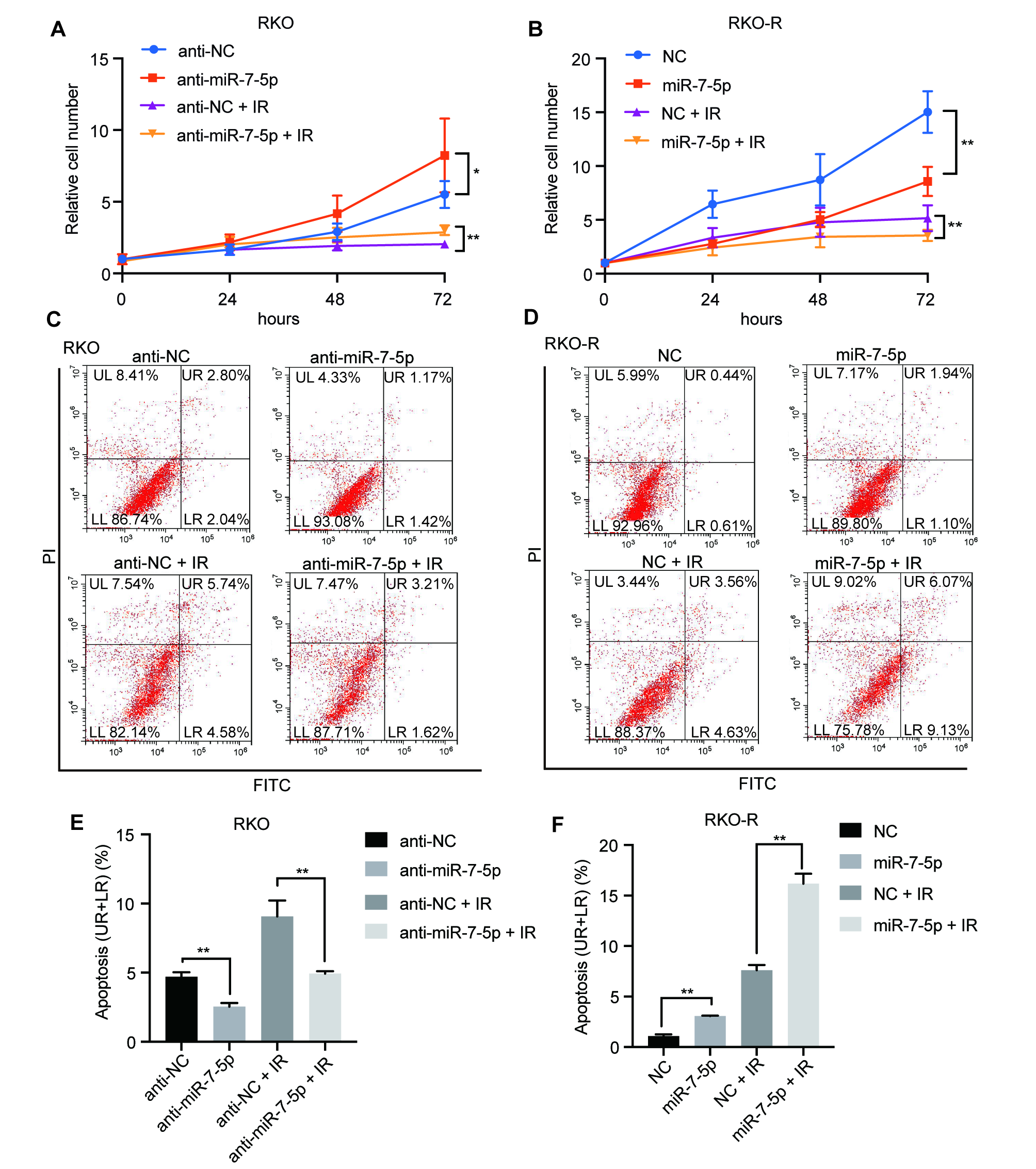


**Figure S4. MiR-7-5p inhibited cell proliferation and promoted radiation-induced apoptosis in RKO and RKO-R under 0 or 4 Gy irradiation. (A)** CCK-8 assays illustrated that the cell proliferation increased in RKO after knockdown of miR-7-5p w/wo 4 Gy irradiation. **(B)** The cell proliferation decreased in RKO-R after overexpression of miR-7-5p w/wo 4 Gy irradiation. **(C-D)** Representative images of apoptosis in RKO and RKO-R after knockdown or overexpression of miR-7-5p in 48h after treatment w/wo 4 Gy irradiation. **(E-F)** The early and late apoptotic cell rates (UR + LR) were quantified. UL, upper left quadrant; UR, upper right quadrant; LL, lower left quadrant; LR, lower right quadrant; w/wo, with or without; Data are presented as mean ± SD; IR, irradiation (4 Gy); *P < 0.05; **P < 0.01.
