# Supplementary material for: MiR-7-5p/KLF4 signaling inhibits stemness and radioresistance in colorectal cancer
Source: Cell Death Discov. 2023 Feb 2;9:42. doi: 10.1038/s41420-023-01339-8 (PMC9894908; doi:10.1038/s41420-023-01339-8)

1. The original full length western blots of Figure 2C


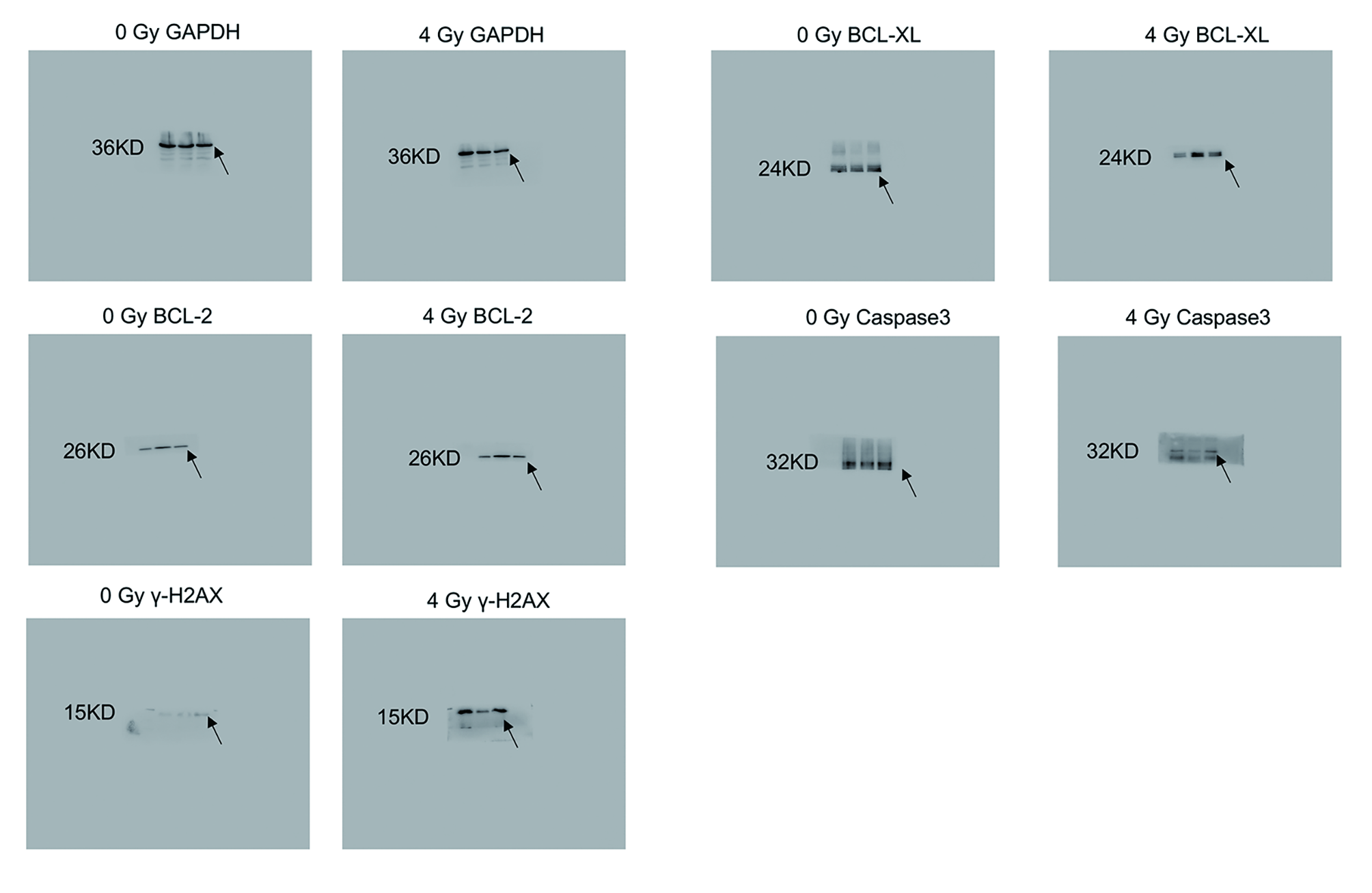


2. The original full length western blots of Figure 3E


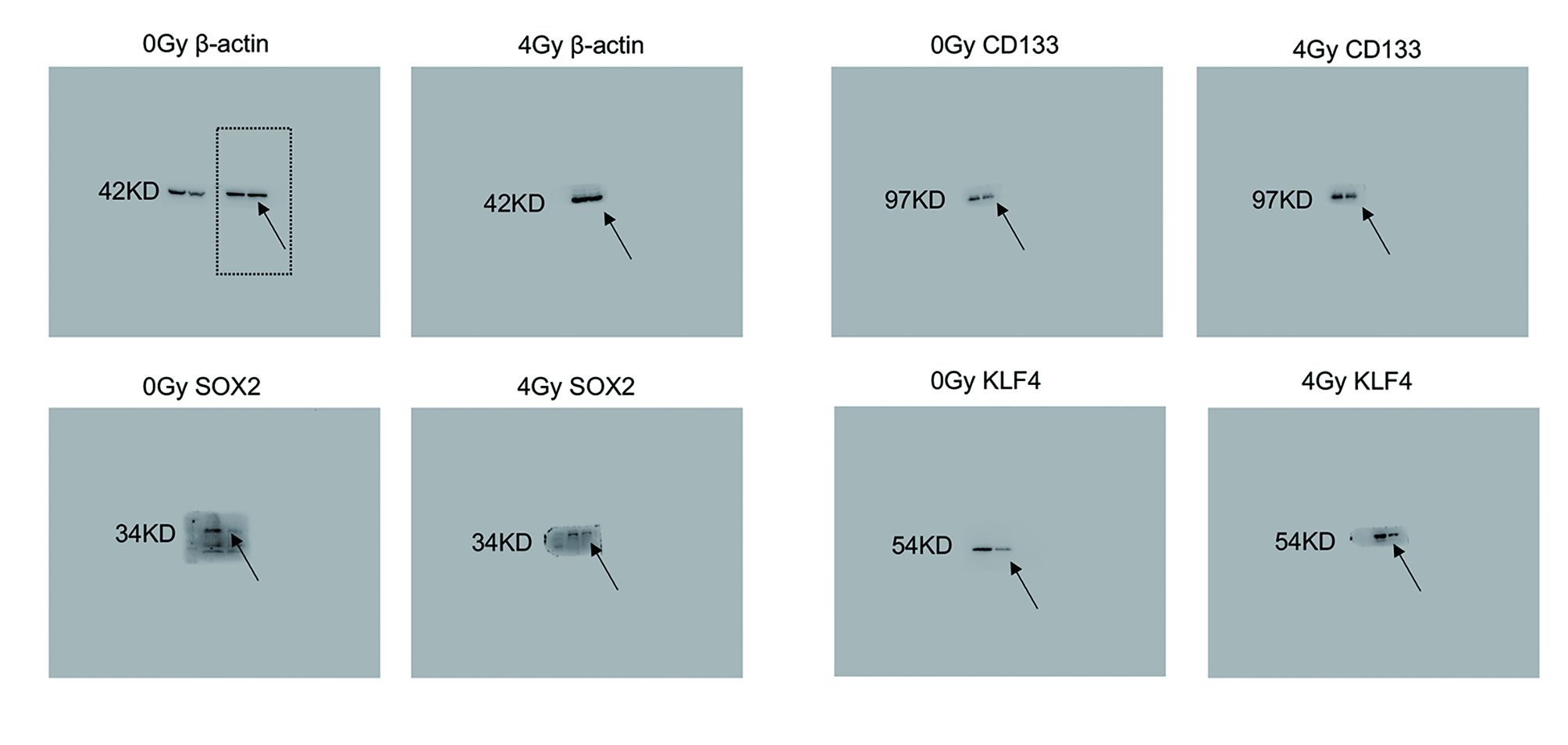


3. The original full length western blots of Figure 3J


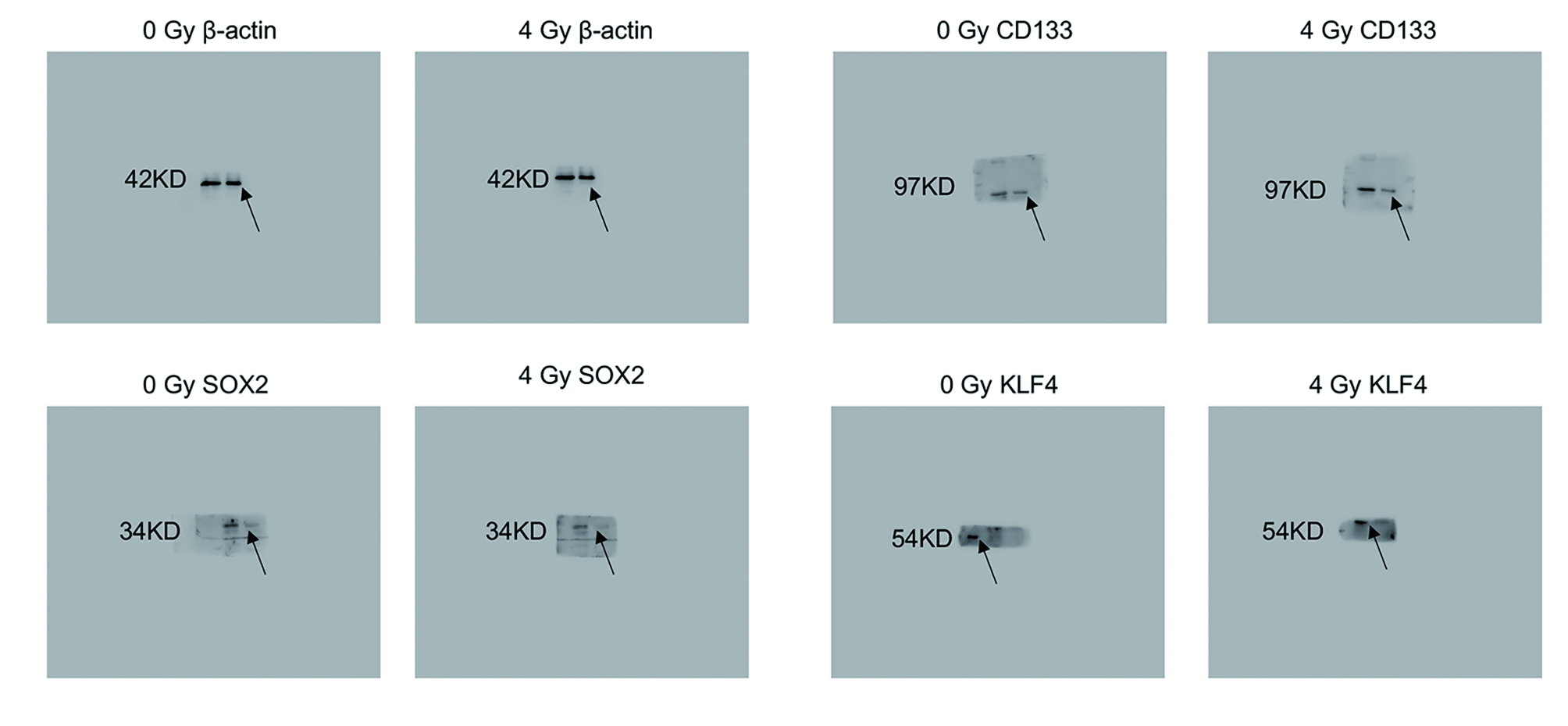


4. The original full length western blots of Figure 4E


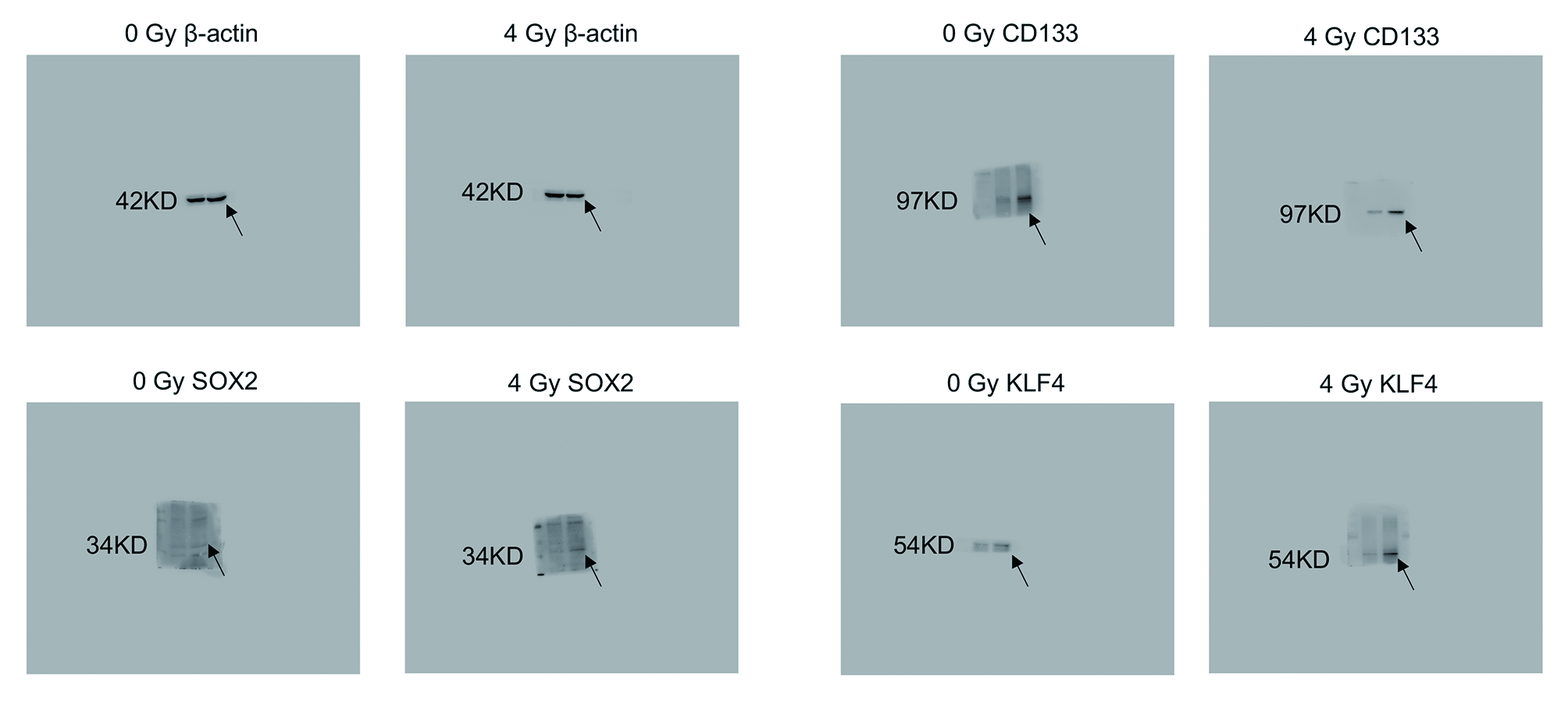


5. The original full length western blots of Figure 5C


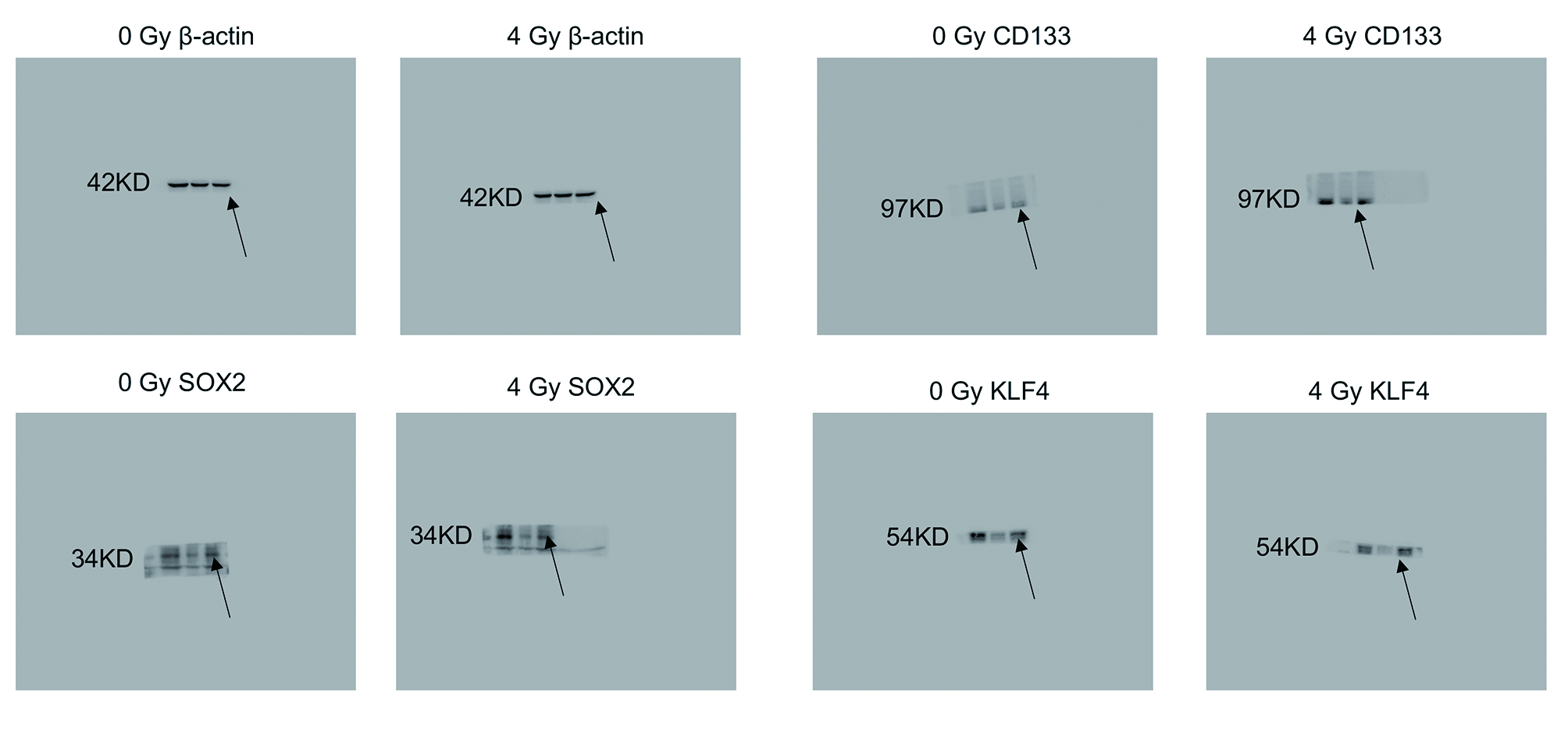


6. The original full length western blots of Supplementary Figure S3D
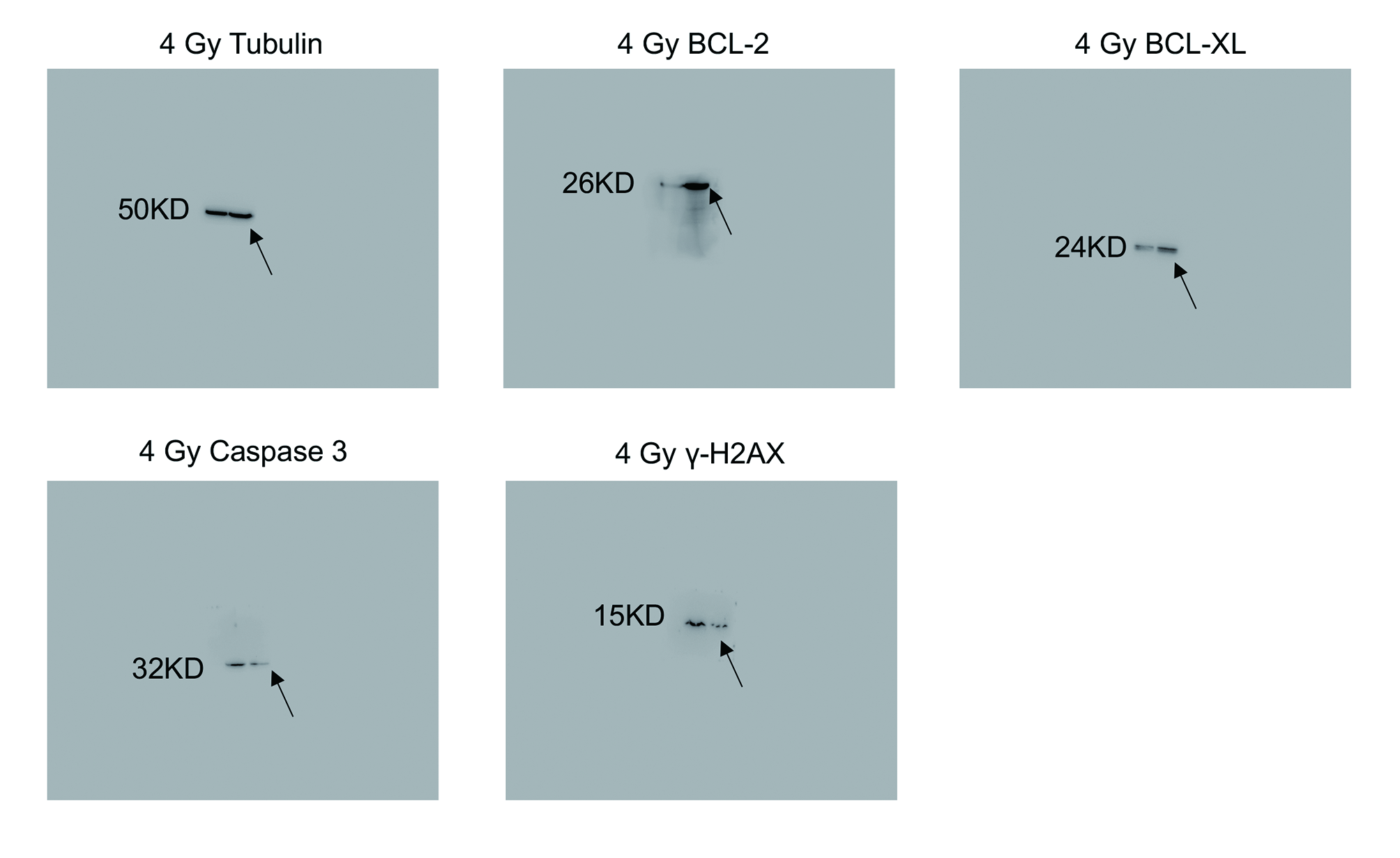

Supplement: Supplementary file 2 — Original Data File [file 41420_2023_1339_MOESM2_ESM.docx]
